# Poxvirus Exploitation of the Ubiquitin-Proteasome System

**DOI:** 10.3390/v2102356

**Published:** 2010-10-19

**Authors:** Michele Barry, Nicholas van Buuren, Kristin Burles, Kelly Mottet, Qian Wang, Alastair Teale

**Affiliations:** Department of Medical Microbiology and Immunology, University of Alberta, Edmonton, T6G 2S2, Canada; E-Mails: njv@ualberta.ca (N.v.B.); burles@ualberta.ca (K.B.); kmottet@ualberta.ca (K.M.); qw1@ualberta.ca (Q.W.); ateale@ualberta.ca (A.T.)

**Keywords:** poxvirus, ubiquitin, F-box, BTB/kelch, RING finger

## Abstract

Ubiquitination plays a critical role in many cellular processes. A growing number of viruses have evolved strategies to exploit the ubiquitin-proteasome system, including members of the *Poxviridae* family. Members of the poxvirus family have recently been shown to encode BTB/kelch and ankyrin/F-box proteins that interact with cullin-3 and cullin-1 based ubiquitin ligases, respectively. Multiple members of the poxvirus family also encode ubiquitin ligases with intrinsic activity. This review describes the numerous mechanisms that poxviruses employ to manipulate the ubiquitin-proteasome system.

## 1. Introduction

Ubiquitin is a 76 amino acid protein that is best known for its role in protein degradation [1], however, the addition of ubiquitin can also serve roles not associated with protein degradation [2]. The post-translational addition of ubiquitin onto proteins occurs through a three step enzymatic cascade [3]. Ubiquitin is initially activated by one of two ubiquitin activating enzymes. Activated ubiquitin is subsequently transferred to a ubiquitin conjugating enzyme. In the final step, a ubiquitin ligase is responsible for the transfer of ubiquitin to the target protein. Proteins can be modified by mono-ubiquitin or poly-ubiquitin [4]. Any one of seven lysine residues present in ubiquitin allows for the formation of ubiquitin chains. Lysine 48 and 63 are the most commonly used. Polyubiquitin chains formed on lysine 48 typically results in degradation through the 26S proteasome [1]. Conversely, polyubiquitin chains formed on lysine 63 tend to alter protein function [2]. More recently, linear ubiquitin has been associated with the regulation of nuclear factor κB, and the ubiquitination of non-lysine residues has also been described [5,6].

The Poxviridae are a large family of viruses that infect a wide range of vertebrates and invertebrates [7]. The best known member of the family is variola virus, the causative agent of smallpox. Global eradication of smallpox was achieved in 1979 through a vaccination program initiated by the World Health Organization (WHO) [8]. Smallpox eradication used vaccinia virus, a close relative of variola virus, as a live vaccine [9]. By virus standards, poxvirus genomes are large ranging in size from 150–300 kbp; encoding upwards of 200 or more open reading frames [7]. Much interest in poxvirus biology stems from the observation that poxviruses employ a vast array of effective immune evasion strategies [10,11]. Additionally, the ease with which recombinant poxviruses are generated has made them attractive viruses for dissecting cellular signaling pathways [10]. Recently, protein ubiquitination has emerged as an important mechanism for the control of protein degradation and function, especially during virus infection [12–16]. In this review we focus on the strategies that poxviruses have developed to exploit the ubiquitin-proteasome system.

## 2. Poxvirus Encoded Ubiquitin

Ubiquitination is a post-translational modification that plays an essential role in many cellular processes [17]. Ubiquitin is a small 76 amino acid protein that is highly conserved in eukaryotes. In fact, only four amino acids differ between yeast, plants and mammalian ubiquitin sequences [18]. Two classes of ubiquitin-encoding genes are present in eukaryotic genomes. These including polyubiquitin genes that encode back-to-back ubiquitin sequences that are cleaved to produce ubiquitin monomers, and ubiquitin-carboxyl extension protein (CEP) fusion genes that encode a single ubiquitin sequence fused to a ribosomal sequence at the C-terminus that is incorporated into the ribosome [19]. Ubiquitin contains seven lysine residues that can be used to build ubiquitin chains [1,17,20]. Traditionally, ubiquitination is associated with protein degradation; however, current evidence indicates that ubiquitination has additional regulatory functions [1,17,20].

**Figure 1:**
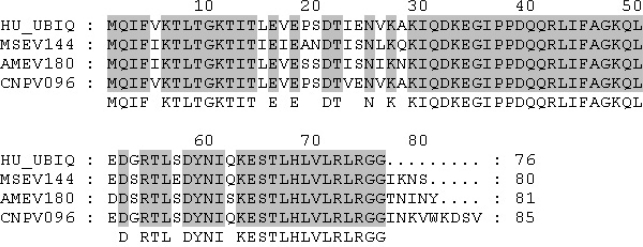
Poxvirus Encoded Ubiquitin. Amino acid sequences of MSEV144, AMEV180, CNPV096 and human ubiquitin were aligned using Clustal W [26,27]. Poxvirus amino acid sequences were obtained from the Poxvirus Bioinformatics Resource Center [28]. Residues representing 100% conservation are shaded.

Ubiquitin homologs have recently been identified in the genomes of poxviruses [21–23]. Two insect poxviruses: *Melanoplus sanguinipes* (MSEV) (Table 1) [21] and *Amsacta moorei* (AMEV) [22], as well as canarypox virus (CNPV), contain virus-encoded ubiquitin homologs [23]. Genomic analysis of MSEV, a poxvirus that infects locusts, identified the open reading frame MSEV144; encoding an 80 amino acid protein that is 86% identical to human ubiquitin (Figure 1) [21]. However, the role that MSEV144 plays during viral infection has not been characterized. BLAST analysis also identified an additional poxvirus-encoded ubiquitin gene AMEV180, in AMEV, a poxvirus that infects moths. AMEV180 is 81 amino acids in length and 89% identical to human ubiquitin (Figure 1) [22]. Sequencing of canarypox, a poxvirus that infects song birds, identified another ubiquitin homolog, CNPV096, in canarypox virus (Figure 1) [23]. At 85 amino acids in length, CNPV096 contains all of the residues required for protein ubiquitination and is 98% identical to human ubiquitin [23]. Interestingly, fowlpox virus (FWPV), a close relative of canarypox virus, contains fragmented remains of a functional ubiquitin gene [24]. MSEV144, CNPV096, and AMEV180 are not part of the polyubiquitin gene class, and do not encode ribosomal peptides at the C-termini. In contrast to eukaryotes, which have multiple copies of ubiquitin-encoding genes, only one copy of each ubiquitin gene is present in the genomes of these viruses [21–23]. Virus-encoded ubiquitin genes have also been identified in *Baculoviridae*, a family of dsDNA viruses that infect insects [25]. Disruption of the ubiquitin gene in *Autographa californica* nuclear polyhedrosis virus (AcNPV) has no effect on virus viability, however, a decrease in virion budding and total infectious particles was observed [25]. Whether the ubiquitin-encoding genes in MSEV, AMEV, and CNPV are required for productive infection or virion budding remains to be determined.

Although most poxviruses do not encode their own ubiquitin genes, ubiquitin is associated with the virion. Proteomic analysis of vaccinia virus indicates that ubiquitin accounts for approximately 3% of total virion protein [29]. Additionally, a lipid-modified form of ubiquitin is associated with several viruses [25,30,31]. For example, baculovirus, African swine fever virus, herpes simplex virus and vaccinia virus incorporate lipid-modified ubiquitin in their envelopes [25,29–31]. Previous analysis of baculovirus AcNPV demonstrated that lipid-modified ubiquitin was present and that ubiquitin was host derived [25]. Scavenging ubiquitin from the host may represent another strategy used by poxviruses to increase the levels of ubiquitin available during infection. Alternatively, lipid-modified ubiquitin may exist in cell membranes for a cellular function, such as autophagosome formation, and the virus simply acquires it passively during envelope acquisition. Whether other poxviruses have lipid-modified ubiquitin incorporated into their envelopes has not been studied.

It seems unlikely that poxviruses would maintain an open reading frame that has no role during infection. To date, the function of the poxvirus encoded ubiquitin sequences has not been determined. Encoding additional pools of ubiquitin could be a mechanism used by entomopoxiruses and canarypox virus to increase efficiency of host cell modulation during infection. It is also possible that these viruses rely heavily on the ubiquitin-proteasome system. For example, it has been shown that members of the Orthopoxvirus family require a functional ubiquitin-proteasome system for productive infection [32,33]. Alternatively, viral-encoded ubiquitin homologs may function to inhibit the ubiquitin proteasome system. The AcNPV-encoded ubiquitin functions as a chain terminator for K48 linked polyubiquitination, the linkage that targets proteins for degradation by the 26S proteasome [34]. As such, it is possible that poxvirus-encoded ubiquitin may also act as chain terminators to inhibit degradation of certain substrates. At present, the reason that only a few members of the poxvirus family encode ubiquitin homologs remains unclear.

## 3. Poxvirus Encoded Ubiquitin Ligases

**Figure 2:**
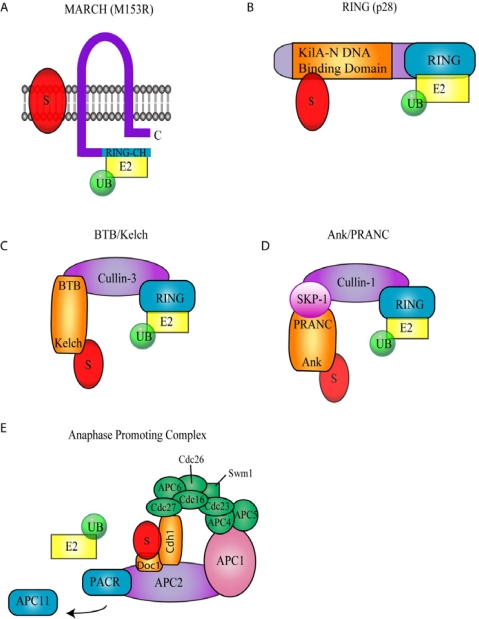
Poxvirus Encoded Ubiquitin Ligases. **(A)** Membrane associated RING-CH (MARCH) ubiquitin ligase. The MARCH ubiquitin ligase, M153R, encoded by myxoma virus contains two transmembrane domains and a C-terminal RING-CH domain. **(B)** p28, a RING Ubiquitin Ligase. p28 contains a C-terminal RING domain and an N-terminal KilA-N DNA binding domain. **(C)** BTB/Kelch ubiquitin ligases. BTB/Kelch proteins interact with cullin-3 through their BTB domain. Potential substrates are likely recruited through the kelch domain. **(D)** Ank/PRANC ubiquitin ligases. Cullin-1 interacts with Skp-1, which in turn interacts with Ank/PRANC proteins. The Ank domain potentially interacts with substrates recruiting them to the cullin-1 ubiquitin ligase. **(E)** Anaphase Promoting Complex (APC). It is hypothesized that PACR displaces APC11 subsequently disrupting APC function.

Poxviruses encode two families of proteins with intrinsic ubiquitin ligase activity; a membrane-associated RING-CH (MARCH) ubiquitin ligase, and a really interesting new gene (RING) finger protein (Figure 2A and B) [35–38].

The MARCH family of proteins contains a modified RING domain (RING-CH) at the N-terminus as well as transmembrane domains that promote localization to membranes (Figure 2A) [39]. Cellular MARCH proteins play an important role in the down-regulation of membrane receptors including MHC class I, MHC class II, and CD4 [39]. In addition to a family of cellular MARCH proteins, MARCH ubiquitin ligases also exist in the genomes of herpesviruses and poxviruses (Table 1) [40]. Infection with myxoma virus (MYXV), a rabbit specific poxvirus that causes myxomatosis, results in reduction of cell surface MHC class I [35,41–43]. The loss of MHC class I upon myxoma virus infection was later associated with ubiquitin ligase activity of the myxoma virus encoded MARCH homolog, M153R [35,41]. In addition to the down-regulation of MHC class I, M153R also reduces cell surface expression of CD95, ALCAM (CD66) and CD4 [35,37,41]. The loss of CD4 by M153R has been well characterized. Upon infection, M153R ubiquitinates the cytoplasmic tail of CD4, leading to its internalization via endocytosis and subsequent lysosomal degradation [37]. Through the action of M153R, myxoma virus induced ubiquitination and degradation of cell surface immune molecules provides an important mechanism for dampening the immune response.

p28 is a virus-encoded RING finger ubiquitin ligase that plays an important role in virulence (Figure 2B) [36,44,45]. p28 is highly conserved among pathogenic poxviruses and is expressed at both early and late times during virus infection (Table 1) [44,45]. The p28 ubiquitin ligase contains two functional domains; an N-terminal DNA binding domain, and a C-terminal RING domain (Figure 2B). The DNA binding domain of p28, referred to as KilA-N, remains largely uncharacterized [46]. This domain is found in a number of large DNA viruses as well as bacteria and bacteriophage [46]. The KilA-N domain plays an important role in the localization of p28 cytoplasmic viral factories [47,48]. In addition to being found in combination with a RING domain, the KilA-N domain is also found independently in some poxviruses. For example, eight KilA-N proteins are encoded in fowlpox virus (FWPV) and 23 KilA-N proteins are encoded in canarypox virus [23]. However, only two proteins in fowlpox virus and canarypox virus combine a KilA-N domain with a RING domain, likely encoding functional ubiquitin ligases [23,24,46].

The C-terminal RING domain of p28 is responsible for ubiquitin ligase activity [36,38]. p28 displays sequence homology to a family of cellular proteins termed Makorin (MKRN), however, this homology is restricted to the RING domain [49]. It has been suggested that the p28 family of poxvirus proteins were acquired through a fusion event of an existing KilA-N domain and a cellular MKRN [49]. Point mutations in the critical conserved residues of the RING domain disrupt ubiquitination [36,38]. Using *in vitro* ubiquitination assays, p28 homologs in ectromelia virus (ECTV), vaccinia virus (VV)-strain IHDW and variola virus (VARV) were shown to function as ubiquitin ligases [36,38]. The p28 ortholog in variola virus, D4R, functions *in vitro* with the ubiquitin conjugating enzymes, Ubc4 and UbcH5c [36]. Work in our laboratory has demonstrated that expression of p28 targets conjugated ubiquitin to viral factories [38]. K48 linked ubiquitin, which is associated with protein degradation, also co-localized at the virus factory with p28 [48]. Given that K48 linked ubiquitin is associated with proteasomal degradation it is likely that p28 plays a role in targeting substrates for degradation. Interestingly, variola virus D4R functions *in vitro* with Ubc13, the only known ubiquitin conjugating enzyme that promotes K63 linkages [36]. In contrast to K48 linkages, K63 linkages are associated with non-proteolytic functions, suggesting that p28 may form K63 linkages during virus infection [36]. To date, no p28 substrates have been identified. However, p28 has been implicated in the inhibition of apoptosis [47,50]. It is therefore tempting to speculate that p28 may be targeting pro-apoptotic proteins for degradation. Since p28 localizes to viral factories, it is likely that potential substrates are located at the viral factory. Additionally, since p28 is expressed early during infection, prior to virus factory formation, p28 may also be responsible for ubiquitinating cytoplasmic substrates.

*In vivo* studies have shown that p28 is a critical virulence factor during ectromelia virus (ECTV) infection [44]. In susceptible strains of mice, ectromelia virus devoid of p28 was extremely attenuated and all mice recovered; this is in sharp contrast to mice infected with wild-type virus, which succumb to infection [44]. Both wild type ectromelia virus and ectromelia virus devoid of p28 replicated equally well in all cell lines tested except for primary peritoneal macrophages [44,45]. Macrophages are thought to be critical for the transport of the virus, suggesting that the ubiquitin ligase activity of p28 plays an important role in peritoneal marcrophages [45]. The role of p28 in virulence and its ability to function as a *bona fide* ubiquitin ligase suggests p28 is ubiquitinating substrates, however these substrates have yet to be identified. Identification of p28 substrates will undoubtedly provide important clues into the role of p28 in virus virulence.

## 4. A Family of Poxvirus Encoded BTB/Kelch Proteins

The BTB domain, also known as the POZ, Bric-a-Brac, Tramtrack, or Broad-complex, is a highly conserved protein-protein interaction motif that is involved in many cellular functions, including transcriptional and cytoskeletal regulation [51–53]. Recently, cellular BTB domain-containing proteins have been shown to function as substrate-specific adaptors of cullin-3 based ubiquitin ligase to target proteins for ubiquitination [54–57]. Unlike the well-characterized SCF (Skp1/Cul1/F-box) and ECS (elonginC/Cul2/SOCS) E3 complexes, in which Skp1/F-box or elonginC/SOCS combine to bridge substrates to cullins, BTB proteins fulfill this function through a single polypeptide containing the BTB domain as a linker to cullin-3 and a substrate-recruiting domain, such as kelch, MATH or Zinc Fingers (Figure 2C) [54–57]. Supporting this, the Skp1 and elonginC proteins display similar three-dimensional structure as the BTB domain [57–59]. The kelch domain consists of multiple repeated kelch motifs, and is thought to mediate protein-protein interactions (Figure 2C) [60].

A large group of BTB/kelch proteins have been identified in most members of the poxvirus family (Table 1) [61]. For example, vaccinia virus encodes three BTB/kelch proteins [62]; cowpox virus (CPXV) encodes six BTB/kelch proteins [63]; ectromelia virus strain Moscow (EVM) encodes four such proteins [64]; while monkeypox virus (MPXV) encodes only one BTB/kelch gene [65] (Table 1). Although the specific roles of the poxvirus BTB/kelch proteins are still unclear, it has been speculated that they may function as cullin-3 substrate-specific adaptors, similar to their cellular counterparts. In agreement with this idea, the BTB domains of ectromelia virus encoded BTB/kelch proteins EVM150 and EVM167 are essential and sufficient for interaction with cullin-3 [66]. Consistently, EVM150 and EVM167 associate with conjugated ubiquitin and Roc1, the RING-finger protein required for an active cullin-3 ubiquitin ligase complex [66]. The other two ectromelia virus encoded BTB/kelch proteins, EVM018 and EVM027, also interact with cullin-3 [67]. Interestingly, EVM004, an ectromelia virus encoded protein containing only a BTB domain, does not interact with cullin-3, Roc1, or conjugated ubiquitin, suggesting that, unlike the other ectromelia virus encoded BTB/kelch proteins, EVM004 may function independently of the ubiquitin-proteasome pathway [67]. The failure of EVM004 to interact with cullin-3 is currently unknown. Together, these findings suggest that poxviruses may employ BTB/kelch-cullin-3 ubiquitin ligase complex as another strategy to manipulate the cellular environment. Alternatively, the poxvirus BTB/kelch proteins may function by simply sequestering cullin-3 to inhibit the cullin-3-based cellular ubiquitin pathway. Given that poxviruses encode multiple BTB/kelch proteins with different kelch regions, it is probable that these viral BTB/kelch proteins function to specifically target different substrates to the cullin-3 ubiquitin ligase for ubiquitination.

The importance of the poxvirus BTB/kelch proteins during virus infection has been studied. Vaccinia virus devoid of the BTB/kelch proteins C2L, F3L or A55R, the orthologs of EVM018, EVM027 and EVM150, respectively, displays an altered viral pathogenesis in the murine intradermal model [68–70]. Deletion of four BTB/kelch genes, D11L, C18L, G3L and A57R, from cowpox virus strain GRI-90 also results in altered host range and attenuated virulence [71]. Additionally, sheeppox virus (SPPV) BTB/kelch gene SPPV-019 has been shown to modulate cellular adhesion and affect virus virulence using a SPPV-019 knock-out virus model [72]. These observations suggest that BTB/kelch proteins function to manipulate the cellular host environment. To date, however, no definite substrates for the poxvirus BTB/kelch proteins have been identified, although several targets for cellular BTB/kelch proteins have been characterized. For example, NRF2, a critical nuclear transcription factor regulating oxidative stress, is degraded by KEAP1/cullin-3 ubiquitin ligase [73,74]. KEAP1 also functions as an IKKβ ubiquitin ligase [75]. Aurora B, a chromosomal passenger protein responsible for the proper progression of mitosis and cytokinesis, is targeted by KLHL21/Cul3 E3 for ubiquitination [76]. Interestingly, the vaccina virus encoded BTB/kelch protein, WR026 (COP-C2L), was recently shown through yeast-2-hybrid screening to interact with cellular crystallin alpha B (CRYAB), a small heat-shock protein [77]. Whether crystalline alpha B can be regulated by WR026 for cullin-3-mediated ubiquitination needs to be investigated. Although the role of poxvirus BTB/kelch proteins is still undefined, many other viruses have evolved mechanisms to specifically recruit cellular proteins to cullin-based ubiquitin ligases [12,14]. Future identification of the substrates targeted by the poxvirus BTB/kelch proteins will provide new insight into the understanding of cellular anti-viral responses.

## 5. Poxvirus Encoded Ankyrin/PRANC Proteins

Ankyrin repeat proteins represent one of the largest families of proteins encoded by poxviruses. The ankyrin repeat consists of a 33 amino acid helix-loop-helix motif with a highly conserved amino acid sequence [78–80]. Ankyrin repeats were first identified in the cytoskeletal structural protein called ankyrin, which contains 24 ankyrin repeats [81]. Since its discovery, the ankyrin repeat has been characterized in a wide variety of cellular proteins, and generally mediates unique protein-protein interactions [78–80]. With the exception of molluscipoxviruses, all other poxvirus families encode a large repertoire of ankyrin proteins (Table 1). The largest family is encoded by canarypox virus and is comprised of 51 ankyrin repeat proteins, representing 21% of the canarypox virus genome [23,82]. Poxviral ankyrin repeat proteins are large proteins, ranging from 400–650 amino acids in length, containing between 5 to 10 ankyrin repeats located at their N-termini. Although the poxviral ankyrin repeat proteins contain no obvious structural domains at their C-termini, many of the proteins display a conserved sequence, which upon closer inspection was shown to resemble the F-box domain that functions in the recruitment of substrates to the cellular SCF (Skp-1, cullin, F-box) ubiquitin ligase complex [82–84]. The poxviral F-box-like domain was later named PRANC (pox protein repeat of ankyrin C-terminus) (pfam.janelia.org/family/PF09372). The SCF complex is a highly conserved ubiquitin ligase involved in regulation of the cell cycle, DNA repair, and innate immunity [17,85,86]. The complex consists of cullin-1, which serves as the molecular scaffold, Roc1, a RING finger ubiquitin ligase, Skp1, the linker protein, and one of over 70 known cellular F-box proteins which function in substrate recruitment (Figure 2D) [84–86]. Cellular F-box proteins consist of N-terminal F-box domains in conjunction with C-terminal protein binding domains such as WD40 repeats or leucine-rich repeats (LRR) [84–86]. The F-box domain consists of a highly conserved 50 amino acid sequence, folding into three alpha-helices, which function to bind the linker protein, Skp1, while WD40 repeats or LRRs function to bind substrates which are subsequently ubiquitinated through the ubiquitin ligase activity of Roc1 (Figure 2D) [59]. Substrates of the SCF ubiquitin ligase complex typically require a phosphorylation event prior to recognition by the substrate adaptor [83–86].

**Figure 3:**
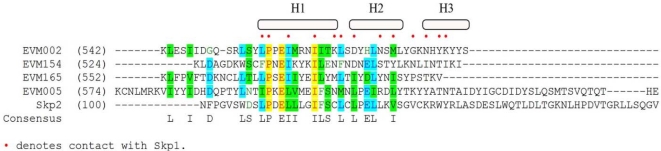
Sequence alignment of ectromelia virus encoded Ank/PRANC proteins with cellular Skp2: AlignX was used to align the C-termini of EVM002, EVM005, EVM154, and EVM165 with the N-terminal F-box domain of Skp2, a cellular F-box protein [89]. Red dots indicate known contact points between Skp2 and Skp1 [59]. H1, H2, and H3 represent alpha-helical secondary structures from Skp2.

Until the recent identification of Ank/PRANC proteins in the parasitoid wasp, *Nasonia*, Ank/PRANC proteins were thought to be unique to poxviruses [87]. The poxvirus Ank/PRANC proteins differ from cellular F-box proteins in two important aspects. Firstly, the C-terminal location of the poxvirus F-box-like domain is unique to this set of proteins, and secondly, the poxvirus Ank/PRANC proteins contain truncated F-boxes (Figure 3) [82,88,89]. The cowpox virus encoded Ank/PRANC protein CP77 contains a PRANC domain that is only 13 amino acids in length and may represent the minimum requirement for interaction with Skp1 [90]. A related family of proteins, the suppressor of cytokine signaling (SOCS)-box family appear in conjunction with ankyrin repeats, and function as substrate adaptor molecules for the ECS ubiquitin ligases [91]. Since the SOCS-box and the F-box share sequence similarity it has been proposed that the poxviral ankyrin/F-box proteins were acquired as SOCS-box proteins that have evolved to regulate the cullin-1 based ligase [82,92]. In addition to poxvirus encoded Ank/PRANC proteins, poxviruses also encode ankyrin only proteins [82,93,94]. These ankyrin-only proteins do not contain PRANC domains, and have been proposed to have arisen from full length Ank/PRANC proteins [82].

Ank/PRANC proteins have been identified in a wide range of poxviruses including vaccina virus, ectromelia virus, cowpox virus and Orf virus. Studies on myxoma virus identified the first interaction between a poxviral Ank/PRANC protein, M-T5, and the SCF complex [95]. MT-5, one of four Ank/PRANC proteins in myxoma virus, co-localizes with cullin-1 in the nucleus and regulates the cell cycle and interacts with Akt [95,96]. Myxoma virus encodes four Ank/ PRANC proteins (M-T5, M148, M149, M150) all of which play a role in myxoma virus virulence [97–99]. Interestingly, M150 co-localized to the nucleus with the p65 subunit of nuclear factor kappa B (NF-κB), suggesting that M150 is involved in inhibition of NF-κB [99]. Each of the five Orf virus encoded Ank/PRANC proteins have been shown to associate with a functional SCF ubiquitin ligase complex, as demonstrated through *in vitro* ubiquitination assays [92]. In the case of the Orf virus proteins, the F-box-like domain was both necessary and sufficient to mediate the interaction with Skp1 and cullin-1 [92]. Similarly, the F-box domains of proteins from ectromelia virus, cowpox virus, and vaccinia virus are also essential for interaction with the SCF complex [89,90,100]. The cowpox virus encoded Ank/PRANC protein, CP77, functions as a host range protein that interacts with the NF-κB transcription factor, p65, to inhibit the transcription of inflammatory cytokines [90,101]. Regulation of the NF-κB signaling pathway by poxviral Ank/PRANC proteins appears to be a common trend. Using a yeast two-hybrid screen, the variola virus encoded G1R Ank/PRANC protein was shown to interact with the NF-κB regulatory protein NFκB1/p105 as well as Skp1 [102]. G1R, and its orthologs in cowpox virus, monkeypox virus, and ectromelia virus (CPXV006, MPXV003, EVM002), bind p105, and inhibit G1R degradation following TNFα stimulus [102]. Additionally, a CPXV006 deletion virus displayed increased release of proinflammatory cytokines in culture, and was slightly attenuated in C57BL/6 mice infected [103].

Although substrates have not been identified for the poxviral Ank/PRANC proteins, it has been hypothesized that the poxvirus Ank/PRANC proteins function as substrate adaptor proteins for the SCF complex. Although it is possible that the poxvirus Ank/PRANC proteins may simply bind and inhibit the SCF complex, this seems unlikely due to the large number of unique Ank/PRANC proteins encoded by poxviruses. For example, fowlpox virus encodes 20 Ank/PRANC proteins, each potentially targeting unique protein(s) for ubiquitination by the SCF complex (Table 1) [24]. Additionally, the ectromelia virus and Orf virus Ank/PRANC proteins have both been shown to associate with functional SCF complexes, suggesting that these proteins do not simply function as inhibitors [89,92]. The identification of substrates recruited to the SCF complex by poxviral Ank/PRANC proteins will be an essential step towards understanding this interesting family.

## 6. Regulation of the APC/C by Poxviruses

The anaphase promoting complex/cyclosome (APC/C) is the largest known cellular ubiquitin ligase complex, composed of at least 12 subunits (Figure 2E) [104]. Since its discovery almost 15 years ago its structure and regulation have proven to be increasingly complex. It is thought that the APC/C complex has evolved from an ancestral SCF-type ubiquitin ligase since the subunits APC2 and APC11 resemble a cullin-family member and RING-type E3 ligase, respectively. APC2 functions as the molecular scaffold, and contains cullin homology and binds to the RING-finger protein APC11 [105]. APC11 has been shown to recruit ubiquitin-conjugating enzymes to the APC/C in order to catalyze the *in vitro* transfer of ubiquitin onto target substrates [106]. Substrates for the APC/C are recognized through the presence of D-box or KEN-box domains, which are recognized by a variety of APC/C components including Cdh1, Cdc20 and Doc1 [107,108]. The APC/C plays a major role in regulation of the cell cycle at several points, and most well known for its ability to degrade securin, a protein that regulates the separation of sister chromatids during anaphase [109].

A family of poxvirus RING-finger proteins was recently identified that contain sequence similarity with the RING domain of the APC/C subunit APC11 (Table 1) [110]. These APC11 homologs were identified in the parapoxviruses, molluscipoxviruses, as well as the crocodilepox and squirrelpox viruses. The poxvirus encoded APC11 homolog from Orf virus, a member of the parapoxvirus family, is the only homolog studied to date and has been named PACR (poxvirus APC/cyclosome regulator) [110]. PACR was shown to co-precipitate with APC/C subunits APC2, APC3 and APC4, and is shown to associate with the APC/C complex in a similar manner to APC11 [110]. However, upon sequence analysis, PACR and the other poxvirus orthologs contain mutations within the RING domain that inhibit the binding of E2 ubiquitin-conjugating enzymes to the complex, and therefore inhibit substrate ubiquitination [110]. It is thought that inhibition of APC/C may prompt cells into S-phase, a stage within the cell cycle where additional cellular factors may be present and contribute to virus replication. Additionally, two of the targets of the APC/C are cellular ribonucleotide reductase and thymidine kinase proteins, proteins that contribute to the free nucleotide pools required for DNA synthesis. Typically poxviruses encode their own thymidine kinase and ribonucleotide reductase genes, however, the viral thymidine kinase and ribonucleotide reductases genes are absent from Orf virus as well as other virus that encode homologs of PARC. In contrast, many viruses that encode their own thymidine kinase genes, lack PACR orthologs. It has been hypothesized that one of the main reasons for encoding APC/C inhibitors is to upregulate cellular thymidine kinase and ribonucleotide reductase genes to enhance free nucleotide pools in poxviruses that lack the ability to promote this themselves.

## 7. Role of the Ubiquitin-Proteasome System During Poxvirus Infection

Poxviruses are renowned for creating an optimal environment for viral replication and propagation [7,11,111]. The ubiquitin-proteasome system, which plays a crucial role in protein degradation and cellular homoeostasis, is an attractive target for virus-encoded effector proteins. The ubiquitin-proteasome system is involved in regulating many important host pathways including antigen presentation, cell cycle progression, signal transduction, and DNA repair [1,17]. Individual interactions between poxviral proteins and the ubiquitin-proteasome system have been characterized [15,16]. The study of the ubiquitin-proteasome system has been aided greatly by the use of chemical proteasome inhibitors. These inhibitors block the catalytic action of the proteasome by preventing the degradation of ubiquitinated proteins and reducing the amount of free ubiquitin available within the cell [112]. Proteasome inhibitors, including MG132, act to reversibly inhibit proteasome action while others, including MG115, lactacystin, and epoxomycin irreversibly inhibit the proteasome [113,114]. Notably, the bortezomib, sold under the trade name Velcade®, and licensed for the treatment of multiple myeloma, is a potent inhibitor of the proteasome [115]. The overall importance of a functioning ubiquitin-proteasome system during poxvirus infection has only recently been investigated [32,33].

It has now been demonstrated that a functioning ubiquitin-proteasome system is vital to a successful infection by members of the *Orthopoxvirus* family [32,33]. In the presence of proteasome inhibitors, poxvirus replication is dramatically impaired [32,33]. Early poxviral gene expression is unaffected while intermediate and late gene expression is greatly reduced through the action of chemically distinct proteasome inhibitors. Viral factories, which normally appear as DNA rich areas in the cytoplasm of infected cells, are unable to form in the presence of proteasome inhibitors. In addition, it has been shown that plasmid replication, which can normally occur during poxvirus replication at viral factories [116], is blocked by the use of proteasome inhibitors [33]. The addition of proteasome inhibitors post-infection indicates that the block affects an early step during poxviral infection but does not affect the entry of poxvirus particles into the cell. Intriguingly, inhibition of the ubiquitin activating enzyme results in a similar phenotype during infection. Since overexpression of ubiquitin is unable to rescue late protein expression, DNA production and the generation of progeny virus, this data suggests that a functional ubiquitin-proteasome system as a whole is required for successful poxvirus infection [33]. Together, these observations indicate that viral DNA replication does not occur upon proteasome inhibition. The lack of viral DNA replication along with the pattern of gene expression seen upon treatement with proteasome inhibitors, points to viral uncoating and DNA replication as the likely candidates for the stage in the poxviral lifecycle actively blocked by proteasome inhibitors [32,33]. Further studies will undoubtedly lead to a greater understanding of the interactions between poxviruses and the ubiquitin-proteasome system and specifically the role of the proteasome during infection.

The dramatic effect of proteasome inhibitors on poxvirus infection, suggests the proteasome may be an attractive target for the development of antivirals. Interestingly, proteasome inhibitors demonstrate an antiviral effect on a wide range of viruses including human immunodeficiency virus [117], influenza virus [118], vesicular stomatitis virus [118], coronavirus [119], human cytomegalovirus [120], respiratory syncytial virus [121], herpes simplex virus [122] and hepatitis B virus [123]. As such, proteasome inhibitors seem to demonstrate antiviral activity though distinct mechanisms among viral species. For example, proteasome inhibitors have been shown to impair entry and RNA synthesis during coronavirus infection [119], inhibit the entry of herpes simplex virus into the nucleus [122], and inhibit influenza and vesicular stomatitis virus replication [118]. However, *in vivo* studies recently conducted have produced mixed results. Treatment with bortezomib results in a decrease of circulating RNA in mice chronically infected with Hepatitis B [123], but proteasome inhibition enhances the disease and mortality in mouse hepatitis coronavirus [119], as well, increasing inflammation and mortality was observed in human respiratory syncytial virus [121]. A possible explanation for the seemingly conflicting results between the *in vitro* and *in vivo* experiments is through modulation of the immune system by proteasome inhibitors. It has been demonstrated that proteasome inhibitors affect antigen processing *in vivo* [124]. While proteasome inhibition may be antiviral, the effects on the immune system caused by proteasome inhibitors may increase susceptibility and mortality in some viral infections. Still, the proteasome remains a possible target for antiviral development against poxviruses and it would be interesting to determine whether proteasome inhibitors are able to inhibit poxvirus disease and mortality *in vivo*.

## 8. Conclusions

Since the first realization that poxviruses encode proteins with intrinsic ubiquitin ligase activity, the field has grown at a fast and exciting pace. It is clear from the current research that poxviruses encode multiple proteins that manipulate the ubiquitin-proteasome system. As discussed here, these strategies include the expression of poxvirus-encoded ubiquitin, ubiquitin ligases, BTB/kelch proteins, Ank/PRANC proteins, as well as inhibitors of the APC/C complex. The presence of multiple poxvirusencoded proteins suggests that poxviruses exploit the ubiquitin-proteasome in order to regulate cellular processes. In support of this, our recent observations indicate that upon infection with vaccinia virus the ubiquitin proteasome system is fully functional [125]. Within the field we have good track record of identifying and characterizing the poxvirus proteins involved in the ubiquitin-proteasome system. However, to date few substrates have been identified. Future studies are likely to focus on the identification of substrates for these viral ubiquitin ligases, as recent advancements in proteomics and mass spectrometry have paved the way to identifying ubiquitinated proteins [126–129]. Future studies will further our understanding of the intricate relationship between poxvirus replication and the ubiquitin-proteasome system.

## Figures and Tables

**Table 1:** Poxvirus encoded modulators of the ubiquitin-proteasome system. Poxviruses are known to encode a number of modulators of the ubiquitin-proteasome system. Included in this table are ubiquitin homologs encoded by poxviruses, as well as MARCH and p28 E3 ubiquitin ligases, BTB/kelch and Ank/PRANC proteins that associate with cellular ubiquitin ligases, and poxvirus APC/cyclosome regulators.

**Involvement in Ubiquitination**	**Genus**	**Virus^a^**	**Gene/Protein**	**Length, aa**	**VBRC accession^b^**
poxvirus-encoded ubiquitin homologs	Avipoxvirus	CNPV-VR111	96	85	VP0043569
	Betaentomopoxvirus	AMEV-Moyer	180	81	VP0037620
	Unclassified Poxviridae	MSEV-Tuc	144	80	VP0038302
MARCH Poxviral E3 Ubiquitin Ligase	Capripoxvirus	GTPV-Pellor	8	162	VP0044818
		LSDV-Nee	10	162	VP0040213
		SPPV-A	8	162	VP0044517
	Leporipoxvirus	MYXV-Lau	M153	206	VP0038581
		RFV-Kas	gp153R	201	VP0038747
	Suipoxvirus	SWPV-Neb	9	155	VP0040564
	Yatapoxvirus	TANV-COD	5	156	VP0067544
		YLDV-Davis	5	156	VP0040054
		YMTV-Amano	4	156	VP0043053
p28 Poxviral E3 Ubiquitin Ligase	Avipoxvirus	CNPV-VR111	205 197	318 275	VP0043678 VP0043670
		FWPV-Iowa	157 150	311 276	VP0037889 VP0037882
	Capripoxvirus	LSDV-Nee	140	240	VP0040345
		SPPV-A	136	240	VP0044645
		GTPV-Pellor	127	240	VP0044947
	Leporipoxvirus	MYXV-Lau	M143	234	VP0038572
		RFV-Kas	gp143R (N1R)	234	VP0038740
	Orthopoxvirus	CMLV-CMS	14R	242	VP0041112
		CPXV-GRI	C7R	242	VP0042678
		ECTV-Mos	12	241	VP0040932
		MPXV-ZAR	D5R	242	VP0040369
		VACV IHD-W	p28	243	^c^
		VARV-BGD75maj	D6R^d^	242	VP0038767
		RPXV-Utr	8	242	VP0041370
	Suipoxvirus	SWPV-Neb	138	246	VP0040694
	Yatapoxvirus	TANV-COD	143R	234	VP0067759
		YMTV-Amano	143R	236	VP0043181
	Unclassified Poxviridae	DPV-W1170_84	154	245	VP0045437
BTB/KELCH proteins associated with cullin-3-based E3 ubiquitin ligase	Capripoxvirus	GTPV-Pellor	16 141 148	562 547 552	VP0044826 VP0044951 VP0044958
		LSDV-NEE	19 144 151	569 547 550	VP0040222 VP0040349 VP0040356
		SPPV-A	16 140 147	569 547 552	VP0044525 VP0044649 VP0044656
	Leporipoxvirus	MYXV-Lau	M014L M140R	517 553	VP0038442 VP0038569
		RFV-Kas	gp013L gp0140R	516 553	VP0038613 VP0038737
	Orthopoxvirus	CMLV-CMS	21L 24L 38L 172R 186R	200 512 480 564 501	VP0041119 VP0041122 VP0041137 VP0041317 VP0041335
		CPXV-GRI	D11L C18L G3L A54R B9R B19R	521 512 485 564 501 557	VP0042668 VP0042689 VP0042703 VP0042838 VP0042849 VP0042686
		ECTV-Mos	18 27 150 165	512 482 563 594	VP0040938 VP0040947 VP0041074 VP0041089
		MPXV-ZAI	D12L D19L C9L	206 107 487	VP0040376 VP0040382 VP0040396
		TATV-DAH68	24 43 181 196	150 480 219 209	VP0052942 VP0052961 VP0053099 VP0053114
		VACV-COP	C2L C5L F3L A55R	512 615 480 564	VP0039555 VP0039551 VP0039572 VP0039751
		RPXV-Utr	15 18 31 162	204 512 480 564	VP0041377 VP0041380 VP0041393 VP0041526
	Suipoxvirus	SWPV-Neb	6 15 136	530 534 574	VP0040561 VP0040570 VP0040692
	Yatapoxvirus	YLDV-Davis	19L 140R	522 570	VP0040068 VP0040192
		YMTV-Amano	19L	524	VP0043062
	Unclassified Poxviridae	DPV-W1170_84	25 159	529 546	VP0045308 VP0045442
ankyrin/PRANC proteins associated with cullin-1-based E3 ubiquitin ligase	Avipoxvirus	FWPV-Iowa	12 14 18 22 26 31 162 218 219 222 227 228 231 232 233 234 240 243 244 246	331 437 700 578 436 341 603 461 434 747 361 525 256 482 512 428 410 262 668 592	VP0037744 VP0037746 VP0037750 VP0037754 VP0037758 VP0037763 VP0037894 VP0037952 VP0037953 VP0037956 VP0037961 VP0037962 VP0037965 VP0037966 VP0037967 VP0037968 VP0037974 VP0037977 VP0037978 VP0037980
	Capripoxvirus	GTPV-Pellor	142 144 145 149	634 498 447 453	VP0044952 VP0044954 VP0044955 VP0044959
		LSDV-Nee	145 147 148 152	634 498 447 489	VP0040350 VP0040352 VP0040353 VP0042090
		SPPV-A	141 143 144 148	631 498 447 484	VP0044650 VP0044652 VP0044653 VP0044657
	Leporipoxvirus	MYXV-Lau	148R 149R 150R 005R (MT-5)	675 490 494 483	VP0038576 VP0038577 VP0038578 VP0038588
	Orthopoxvirus	CMLV-CMS	3L 4L 177L 197R	585 672 564 783	VP0041099 VP0041101 VP0041325 VP0041349
		CPXV-GRI	D3L D4L D8L (CP77) C1L C11L B3R B16R B18R K1R I2R I3R	586 672 661 437 614 558 574 795 581 672 586	VP0042660 VP0042661 VP0042665 VP0042672 VP0042682 VP0042843 VP0042856 VP0042858 VP0042863 VP0042868 VP0042869
		ECTV-Mos	2 5 154 165	587 650 564 594	VP0040921 VP0040924 VP0041078 VP0041089
		MPXV-ZAR	B5R J1R N4R B17R	561 587 437 793	VP0040530 VP0040553 VP0040552 VP0040542
		TATV-DAH68	220 187 18 6	640 558 661 627	VP0053138 VP0053105 VP0052936 VP0052924
		VACV-Cop	B18R C19L B4R	574 259 558	VP0039778 VP0039532 VP0039761
		RPXV-Utr	180 178 166	791 574 558	VP0041544 VP0041542 VP0041530
		VARV-BDG75maj	B5R G1R B16R B18R	558 585 574 787	VP0038933 VP0039159 VP0038944 VP0038946
	Parapoxvirus	ORFV-NZ2	8 123 126 128 129	516 525 497 500 520	VP0047660 VP0047777 VP0047780 VP0047782 VP0047783
	Suipoxvirus	SWPV-Neb	141 142 143 144	635 485 430 493	VP0040697 VP0040698 VP0040699 VP0040700
	Yatapoxvirus	YLDV-Davis	148R 147R 146R 11L	476 491 473 637	VP0040200 VP0040199 VP0040198 VP0040060
		YMTV-Amano	11L 146R 147R 148R	637 356 497 483	VP0043056 VP0043184 VP0043185 VP0043186
	Unclassified Poxviridae	DPV-W1170_84	164 163 162 160 19	493 483 501 641 643	VP0045447 VP0045446 VP0045445 VP0045443 VP0045302
poxvirus APC/cyclosome regulators	Molluscipoxvirus	MOCV-st1	026L	83	VP0038021
	Parapoxvirus	BSPV-AR02	13	93	VP0043354
		ORFV-NZ2	14	93	VP0047667
	Unclassified Poxvirdae	CRV-ZWE	47	81	VP0066074
		SPV	A11L	86	DQ377804^e^

a Representative strains were chosen for each individual virus, and the viruses are abbreviated: Canarypox virus (CNPV), Fowlpox virus (FWPV), Goatpox virus (GTPV), Lumpy skin disease virus (LSDV), Sheepox virus (SPPV), Myxoma virus (MYXV), Rabbit fibroma virus (RFV), Molluscum contagiosum virus (MOCV), Camelpox virus (CMLV), Cowpox virus (CPXV), Ectromelia virus (ECTV), Monkeypox virus (MPXV), Taterapox virus (TATV), Vaccinia virus (VACV), Variola virus (VARV), Bovine papular stomatitis virus (BPSV), Orf virus (ORFV), Swinepox virus (SWPV), Tanapox virus (TANV), Yaba-like disease virus (YLDV), Yaba monkey tumor virus (YMTV), *Amsacta moorei* enomopoxvirus (AMEV), *Melanoplus sanguinipes* entomopoxvirus (MSEV), Mule deer poxvirus (DPV), Nile crocodile poxvirus (CRV), Squirrel poxvirus (SPV).

b VBRC accession numbers were obtained from the Poxvirus Bioinformatics Resource Center [28].

c The complete VACV-IHD-W genome has not been published and an accession number is not available.

d D6R is also known as D4R, B5R or B6R, depending on the strain of VARV.

e The SPV genome is not available in the Poxvirus Bioinformatics Resource Center so the accession number from GENBANK was used.
